# Successful Treatment of Hematopyometra in One Horn of a Didelphys Uterus While Maintaining a Viable Pregnancy in the Other Horn

**DOI:** 10.1155/crog/9982018

**Published:** 2026-02-11

**Authors:** Stephanie Matsuura, Selin Kutlu, Alyssa Malley

**Affiliations:** ^1^ Department of Obstetrics, Gynecology, and Women’s Health, University of Hawai’i, John A. Burns School of Medicine, Honolulu, USA

**Keywords:** didelphys uterus, hematopyometra, pregnancy, uterine anomaly

## Abstract

Congenital uterine anomalies are rare, with didelphys uterus being one of the least common. Although generally asymptomatic, it can be associated with infertility, dysmenorrhea, and various pregnancy complications including spontaneous abortion and preterm delivery. We present the case of a 29‐year‐old gravida one para zero female at 6 weeks and 4 days gestational age who presented with pelvic pain during pregnancy. She was incidentally found to have a didelphys uterus during evaluation. She was diagnosed with a hematopyometra in the right horn, likely the cause of her pelvic pain, while carrying a viable pregnancy in the left horn. She was successfully treated with antibiotics and hysteroscopy with dilation and curettage. The remainder of her pregnancy was relatively uncomplicated until delivery and her postpartum course was uneventful. This case demonstrates a rare but possible pregnancy complication in a patient with didelphys uterus as well as successful management of that complication in pregnancy.

## 1. Introduction

Congenital uterine anomalies comprise a variety of abnormalities of the female reproductive tract. Didelphys uterus or “double uterus” is one of the least common congenital uterine abnormalities, accounting for 8%–10% of all uterine anomalies. It is caused by the incomplete fusion of the mullerian ducts in utero, resulting in two uterine horns, each with their own cervix [1–4]. Didelphys uterus is often asymptomatic, so delay in diagnosis is common. Diagnosis is typically made with transvaginal ultrasound, hysteroscopy, and/or MRI [3].

Didelphys uterus is associated with infertility, dyspareunia, dysmenorrhea, and various pregnancy complications including spontaneous abortion, breech presentation, cesarean delivery, and preterm delivery [1–4]. Pregnant patients with known didelphys uterus should be counseled on the increased risk of pregnancy complications and supervised closely throughout their pregnancy.

Pelvic infection in pregnancy is rare due to the presence of the mucus plug and decidua, which typically prevent ascending bacteria from entering the uterine cavity. However, prior to 12 weeks gestation, the mucus plug and decidua have not fully formed. In addition, having a mullerian anomaly can be a risk factor for pelvic infection [5]. This case describes a patient who was treated for hematopyometra in the first trimester in one uterine horn, while continuing to carry a pregnancy in the other.

## 2. Case

**Figure 1:**
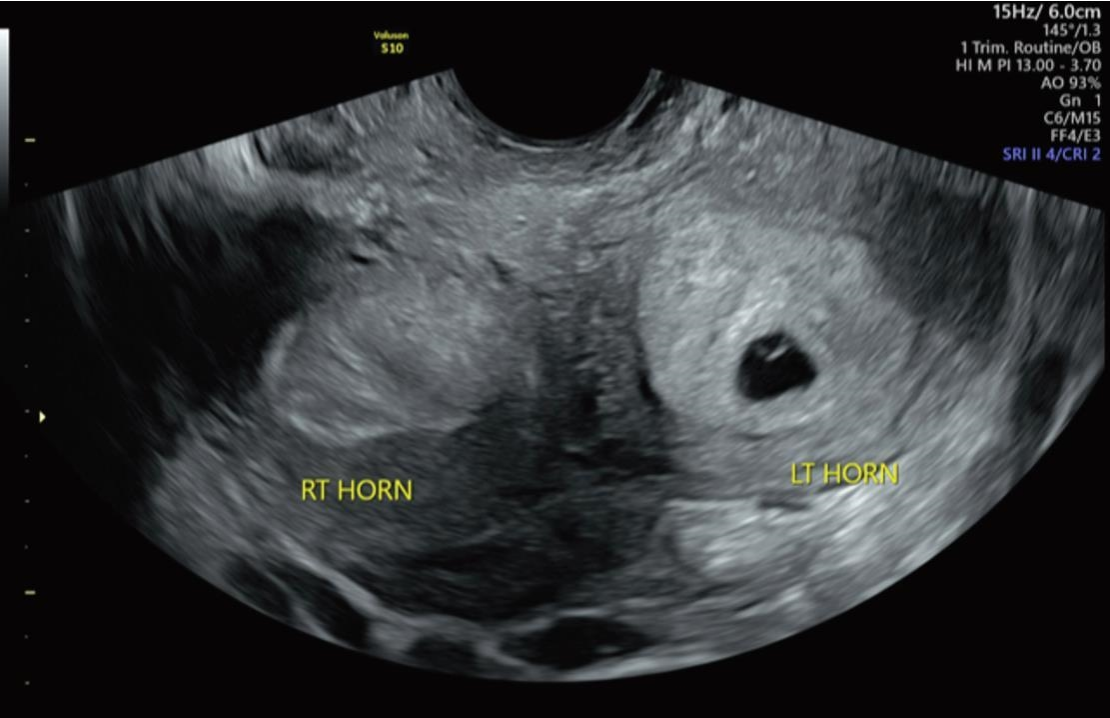
Transvaginal ultrasound showing both uterine horns.

**Figure 2:**
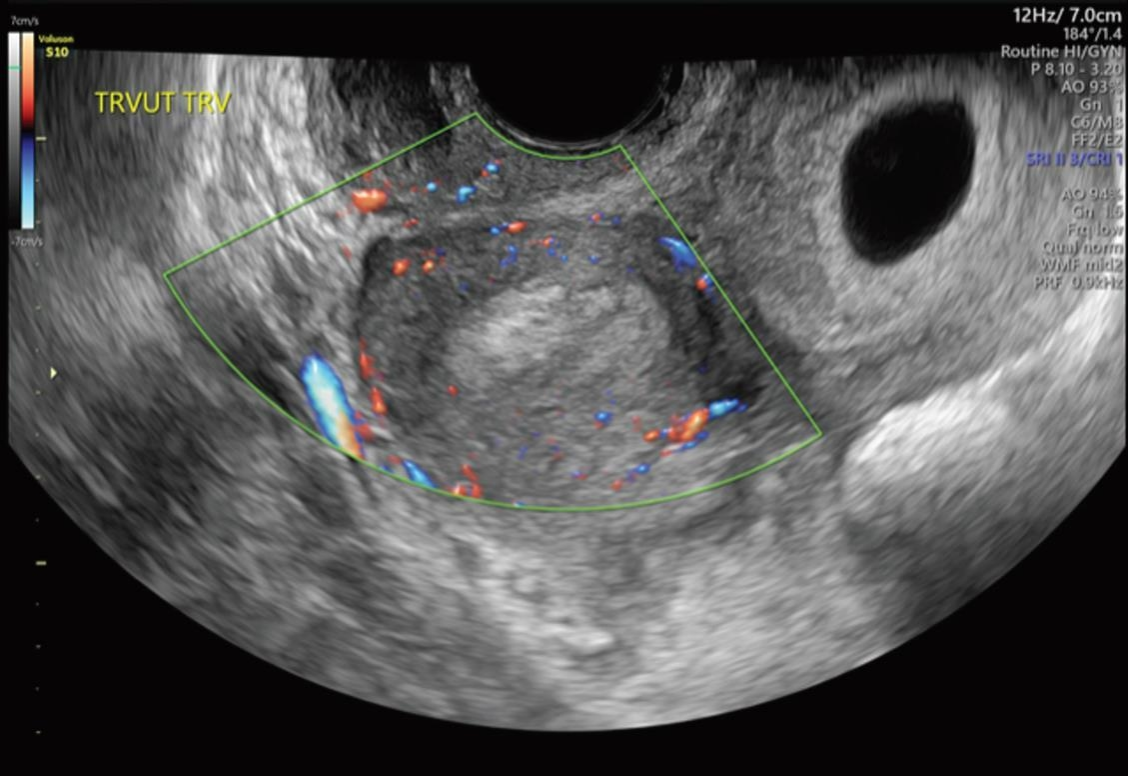
Transvaginal ultrasound demonstrating heterogenous material in right uterine horn.

A 29‐year‐old gravida one para zero female at 6 weeks 4 days gestational age by last menstrual period consistent with ultrasound presented to the emergency department for pelvic pain and intermittent vaginal bleeding for 2 weeks. Prior imaging had demonstrated either a bicornuate or didelphys uterus with a pregnancy in the left horn. She was afebrile with normal vital signs. On physical examination, she endorsed lower abdominal tenderness with palpation and cervical motion tenderness on bimanual exam. Laboratory tests were significant for leukocytosis at 28 × 10^3^ cells/*μ*L. A recent gonorrhea and chlamydia nucleic acid test was negative. She was undergoing treatment for bacterial vaginosis with metronidazole at this time. Formal ultrasound performed in the emergency department demonstrated an intrauterine pregnancy with cardiac activity in the left horn (Figure 1) and heterogeneous material in the right horn (Figure 2). Speculum exam confirmed two cervices, with a small cervical os on the right with active slow leakage of purulent dark red blood. She was admitted to the hospital and started on IV cefoxitin 2 gm every 6 hours, oral azithromycin 1 gm once, and oral metronidazole 500 mg twice a day for suspected pelvic infection, or hematopyometra, of the right horn in pregnancy.

The following day, the patient noted continued lower abdominal discomfort and the white blood cell count remained elevated at 21 × 10^3^ cells/*μ*L. After discussion with the patient, the decision was made to proceed with surgical management of the infection. She was subsequently taken to the operating room for a hysteroscopy with dilation and curettage of the right uterine horn for evacuation of the suspected hematopyometra. Transabdominal ultrasound was used to confirm entry into the proper horn for the duration of the case. Purulent drainage was noted from the right horn during dilation. Suction cannula was used to evacuate the right uterus, which was ultimately confirmed with hysteroscopy. The patient’s recovery was uneventful and IV cefoxitin and oral metronidazole were continued. Patient denied pain on postoperative day one. Her vitals remained within normal limits and she was afebrile. She was discharged home on oral azithromycin 250 mg daily and metronidazole 500 mg twice a day for a total of 14 days of antibiotics.

Her pregnancy remained uncomplicated until she was noted to have severe range blood pressures at 36 weeks gestation. She was subsequently induced for preeclampsia with severe features by blood pressure criteria. She ultimately underwent cesarean delivery for failed induction of labor and fetal intolerance to labor remote from delivery. She recovered well postpartum and was discharged home on her fourth postoperative day.

## 3. Discussion

Upper genital tract infection is uncommon in pregnancy; however, it can be more common in the first trimester, as the mucus plug and decidua have not fully developed. The accumulation of purulent bloody fluid in the uterine cavity, or hematopyometra, can be a complication of upper genital tract infection [5, 6]. This condition is very rare with an incidence of 0.01%–0.5% [7]. It can occur with cervical obstruction, which can be more common in patients with a mullerian anomaly. Timely diagnosis of pyometra is important to prevent complications, such as uterus perforation or sepsis [6]. However, diagnosis can be challenging due to the rarity of the condition and the nonspecific presentation, which typically includes lower abdominal pain, purulent vaginal discharge, and vaginal bleeding. Early diagnosis and treatment are crucial to reduce patient morbidity and mortality [7, 8].

To our knowledge, this is one of the first cases of a pregnant patient with a didelphys uterus complicated by a hematopyometra. Not only did this patient have a rare uterine anomaly, but she also had a rare uterine infection in one uterine horn while maintaining a viable pregnancy in the other horn. Early diagnosis reduced the patient’s risk of complications, which may have required more intensive medical treatment and potential additional surgery to evacuate the infection. In addition, it may have affected her pregnancy. After diagnosis, the patient was successfully treated with minimally invasive surgery and a prolonged course of antibiotics. Alternate treatment options could have included expectant management, although this would generally not be recommended in the setting of infection, or antibiotics alone, as described in a similar case [9]. It was felt that prompt evacuation of the infection would be the best option to maintain a healthy pregnancy. She subsequently had a relatively uneventful remainder of her pregnancy, which ended in cesarean delivery of a healthy child and an uncomplicated postpartum period.

This report is a reminder that patients with mullerian anomalies can experience unique complications in pregnancy. Although uncommon, pelvic infection is important to keep on the differential in a patient with pelvic pain in early pregnancy, and can generally be confirmed with physical exam and prompt imaging.

## Funding

No funding was received for this manuscript.

## Consent

Verbal informed consent was obtained from the patient and the patient has been sufficiently anonymized according to ICMJE guidelines. IRB approval was not obtained because the patient had verbally consented to the case report and the patient was sufficiently anonymized.

## Conflicts of Interest

The authors declare no conflicts of interest.

## Data Availability

The data used to support the findings of this study are included in the article.
